# Home birth and barriers to referring women with obstetric complications to hospitals: a mixed-methods study in Zahedan, southeastern Iran

**DOI:** 10.1186/1742-4755-9-5

**Published:** 2012-03-20

**Authors:** Mahmoud Ghazi Tabatabaie, Zahra Moudi, AbouAli Vedadhir

**Affiliations:** 1 Department of Demography & Population Studies, Faculty of Social Sciences, University of Tehran, Tehran, 14395-773, Iran; 2 Faculty of Nursing and Midwifery, Zahedan University of Medical Sciences, Zahedan, Iran; 3 Department of Anthropology, Faculty of Social Sciences, University of Tehran, Tehran, 14395-773, Iran

**Keywords:** home birth, referral system, delay, maternal mortality, women, complication

## Abstract

**Background:**

One factor that contributes to high maternal mortality in developing countries is the delayed use of Emergency Obstetric-Care (EmOC) facilities. The objective of this study was to determine the factors that hinder midwives and parturient women from using hospitals when complications occur during home birth in Sistan and Baluchestan province, Iran, where 23% of all deliveries take place in non- hospital settings.

**Methods:**

In the study and data management, a mixed-methods approach was used. In the quantitative phase, we compared the existing health-sector data with World Health Organization (WHO) standards for the availability and use of EmOC services. The qualitative phase included collection and analysis of interviews with midwives and traditional birth attendants and twenty-one in-depth interviews with mothers. The data collected in this phase were managed according to the principles of qualitative data analysis.

**Results:**

The findings demonstrate that three distinct factors lead to indecisiveness and delay in the use of EmOC by the midwives and mothers studied. Socio-cultural and familial reasons compel some women to choose to give birth at home and to hesitate seeking professional emergency care for delivery complications. Apprehension about being insulted by physicians, the necessity of protecting their professional integrity in front of patients and an inability to persuade their patients lead to an over-insistence by midwives on completing deliveries at the mothers' homes and a reluctance to refer their patients to hospitals. The low quality and expense of EmOC and the mothers' lack of health insurance also contribute to delays in referral.

**Conclusions:**

Women who choose to give birth at home accept the risk that complications may arise. Training midwives and persuading mothers and significant others who make decisions about the value of referring women to hospitals at the onset of life-threatening complications are central factors to increasing the use of available hospitals. The hospitals must be safe, comfortable and attractive environments for parturition and should give appropriate consideration to the ethical and cultural concerns of the women. Appropriate management of financial and insurance-related issues can help midwives and mothers make a rational decision when complications arise.

## Background

In 2008, 358,000 maternal deaths occurred worldwide, which was a 34% decline from the level of 1995 [1]. Notwithstanding this overall decline, 99% of these deaths occurred in developing countries. Over 25% of all maternal deaths during delivery and within the first 24 hours after delivery occur in developing countries. 50% of maternal deaths occur within the first week after delivery [2] due to delivery complications [3]. While these complications are not generally predictable [4,5] and arise even in well-nourished, well-educated women who receive sufficient prenatal and delivery care, the majority of maternal deaths can be prevented by timely medical intervention during labour, delivery and the immediate postpartum period [6]. Therefore, to prevent maternal death, appropriate emergency care must be available [7].

Thus, because predicting which individual woman will develop a life-threatening complication is difficult, all pregnant women should have access to a qualified provider of delivery health care, and adequate services should be available at the referral level [8]. A brief review of the literature suggests that one factor that contributes to high maternal mortality in developing countries is a delay in accessing EmOC facilities when life-threatening complications arise during childbirth. As Thaddeus and Maine (1994) observed, this delay can be classified into three levels (the three-level model): Delay in making the decision to seek care; delay in arriving at a health-care facility; and delay in receiving adequate treatment. This conceptual framework addresses the factors that appear in the interval between the onset of an obstetric complication and its resolution, e.g., seeking care for obstetric emergencies outside the home [5].

In Iran, the maternal mortality rate declined sharply between 1989 and 1997, from 91 deaths per 100,000 live births to 37.4 deaths per 100,000. The maternal mortality rate did not decrease from 1996 to 2004, remaining at approximately 37.4 deaths per 100,000 live births. Since 2004, the maternal mortality rate has declined slowly. Currently, the rate is approximately 30 deaths per 100,000 live births [1]. To decrease maternal mortality, Iran's Ministry of Health and Medical Education has implemented several strategies and policies, including the "safe motherhood package". Because the vast majority of complications and deaths occur during and immediately after delivery and are due to sudden and unexpected complications [8], the ministry has emphasised providing comprehensive EmOC and hospital birth. The situation is worse in some areas of Iran such as Sistan and Baluchestan province, where 10.8% and 19.5% of all deliveries in urban and rural areas, respectively, occur at home, in comparison to 0.8% and 5.4% for the entire country [9]. The objective of this study was to determine the factors that hinder midwives and parturient women from using hospitals when complications occur during home births in Zahedan.

### Setting

The data presented in this paper were collected in Zahedan, the capital and most populous city of Sistan and Baluchestan province in southeastern Iran, where 23% of all deliveries occur in non-hospital setting [10]. Sistan and Baluchestan province is located on the border with Pakistan and numbers among Iran's poorest provinces. The population of Zahedan consists of two ethno-linguistic groups, the Baluch and the Sistanis, who are the province's indigenous inhabitants. The Baluch, who live mainly in Pakistan, Iran and Afghanistan, are in the majority in Zahedan and are typically Sunni Muslim, Islam's largest religious group. The non-Baluch inhabitants of the province are mainly Shiite. In 2010, the population growth rate and the total fertility rate in Zahedan were 2.5% and 3.6% for each woman of reproductive age in 2010, respectively. Zahedan has four comprehensive EmOC services and two birth centres. These centres are located in the city's suburbs and staffed 24 hours a day by medically certified midwives without the presence of obstetricians or other physicians. The midwives assist only with normal vaginal delivery. They are not allowed to administer antibiotics and anticonvulsants or manually remove a trapped placenta. In the event of the latter scenario, the midwives refer the patients to a hospital. Despite the availability of these facilities, 12 per cent of women still choose to deliver their babies at home with the assistance of a traditional birth attendant, a trained birth attendant or a biomedically educated midwife. Biomedically educated midwives are allowed to run private offices and assist with normal vaginal birth in homes [10].

## Methods

Given the conceptual and contextual complexity of the issues involved, this study uses a mixed-methods (MM) research approach. MM may be most simply defined as a research design in which qualitative and quantitative approaches are used in determining question types, research methods, data collection and analysis procedures, and inference [11]. In the quantitative phase, the analysis was performed on the existing metric data that are routinely collected by the health-care sector. These data were used to calculate the UN process indicators for emergency obstetric services. The UN agencies UNICEF, WHO, and UNFPA in collaboration with the Averting Maternal Death and Disability (AMDD) programme developed the UN process indicators to measure the availability, use and quality of emergency obstetric care (EmOC) [12-14]. In addition to basic EmOC (administering parenteral antibiotics, oxytocics, anticonvulsants for preeclampsia and eclampsia, manually removing the placenta or other retained tissues and assisting with vaginal delivery), EmOC should offer Caesarean section and blood transfusion [12]. The UN standard requires that a service have been provided for a period of at least the past three months [15]. The definition and minimum acceptable levels of the indicators are shown in Table 1.

**Table 1:** The three UN EmOC process indicators and their recommended levels

EmOC indicators	Description	Acceptable level
1. Availability of EmOC	Number of facilities that provide EmOC, defined as providing 8 medical services in the last 3 months	At least 1 comprehensive EmOC facility per 500,000 people and 4 basic EmOC facilities per 500,000 people
2. Proportion of all births in EmOC facilities	proportion of all expected births in the population that took place in an EmOC facility	At least 15% of all births in the population take place in either basic or comprehensive EmOC facilities
3. Caesarean section as a percentage of all births	Caesarean section as a proportion of all births in the population	At least 5% and no more than 15% of all births

Abbreviation: EmOC, emergency obstetric care

Source: AMDD working group on indicators, 2002.

The qualitative phase of the study includes collection and analysis of seventeen interviews with informant midwives and skilled attendants and twenty-one in-depth interviews with Baluch mothers and their relatives. These interviewees were recruited in such a way that their experiences can help us to answer the research questions. All qualitative data were managed and interpreted thematically while drawing upon the strategies and principles of qualitative data analysis, including Strauss and Corbin's coding algorithm for grounded theory.

### Participants and data collection

The qualitative data presented in this study were collected from mothers and midwives who live and work in Zahedan. Interviews were conducted with fourteen biomedically educated midwives who had private offices in Zahedan and assisted with home birth and with three traditional birth attendants (TBA). Participants were recruited according to the research aims. First, informants were selected among midwives with home birth and referral experience. Among these informants, four midwives worked both in state hospitals and private clinics and one worked only in a private clinic. Second, three TBAs were interviewed, of whom one was trained and worked under government supervision. The two other TBAs were untrained and worked independently. Additionally, twenty-one Baluch mothers were interviewed. Each of these mothers had given birth at home and some of them had experienced delay in the use of EmOC. After asking for consent, the midwives and TBAs interviewed gave the mothers' names and telephone numbers to the researcher. The researcher telephoned each mother to establish the time and place of the interviewees. The interviews ceased when the researchers were convinced that no new information of value to the project would emerge [16]. As mentioned above, the qualitative data were gathered through in-depth, semi-structured (open-ended) interviews in the homes of the mothers and TBAs and the private offices of the biomedically educated midwives. The interviews lasted from 60 to 120 minutes. In the interviews, the following issues were discussed: a) barriers that prevent the mothers and their families from accepting a referral and b) barriers that prevent the midwives from proposing a referral. All interviews were conducted in Persian by one of the authors. All interviews were audiotaped, transcribed verbatim, and analysed.

### Analysis

A sequential MM approach was designed to manage and analyse the evidence and information collected [11,17]. In the qualitative phase, the analysis was performed on the existing data and indicators from the health-care sector using the UN indicators for EmOC services and the UN minimum acceptable levels of EmOC [12,13]. In the qualitative phase, the data were analysed according to various strategies and principles of qualitative data analysis, including grounded theory. The essence of grounded theory is inductive-deductive interplay beginning not with a hypothesis but with data collection and then allowing relevant ideas to develop [18]. In this view, Strauss and Corbin's coding offered a more comprehensive understanding of the reasons why Baluch women resist referral and why midwives were reluctant to refer mothers to hospitals at the first signs of birthing difficulty. The first step was a line-by-line reading and open coding of the data. Data coding has been performed by the main researcher (a midwife), a counselling psychologist and an anthropologist. Then, two midwives (one biomedically educated and one a TBA) were given the transcript and codes for confirmation. Next, categories were determined by a constant comparison of the codes [19]. Two midwife members of Zahedan's maternal mortality committee were also asked to confirm the results and comment. Two main categories could be recognised: first, mothers and family members who cited expense, socio-cultural and familial reasons, and perceived quality of care as barriers; second, midwives who cited the professional prestige reasons and lacked communication skills required to persuade mothers to accept referral.

### Ethical approval

This study was initially approved by the ethical committee of Shaheed Beheshti University of Medical Sciences, Tehran, and the relevant regional authorities of Sistan and Baluchestan province in 2010. The authors of this study obtained the formal permission of all participants to perform the interviews. The voluntary nature of participation in this study was emphasised. Confidentiality was guaranteed regarding the identity and other personal information of all interviewees.

## Results

### Availability and economic accessibility of EmOC

Table 2 presents the availability of EmOC according to a needs-assessment study performed in Zahedan in 2011. As the table shows, Zahedan is not short of comprehensive EmOC. However, the city lacks basic EmOC, a shortage partly covered by two suburban Safe Delivery Centres.

**Table 2:** EmOC Availability in Zahedan, Iran

Population Size	Current availability		Recommended number	
	
	*Basic*	*Comprehensiv*	*Basic*	*Comprehensiv*
613,572	-	4	4.9 *	1.2**

* Standard: 4 basic per 500,000 (UNICEF UNFPA WHO 1997, UNFPA report 2002).

**Standard: 1 comprehensive per 500,000 (UNICEF UNFPA WHO 1997, UNFPA report 2002).

Source: MHO, Zahedan, 2011.

For a number of Zahedan residents, the four comprehensive EmOC facilities are not economically accessible. As described in the literature [5,20], "economic accessibility" refers to the relationship between a family's financial capability and the costs of obstetric care. Directly or indirectly, economic inaccessibility significantly delays the decision to use a delivery facility. In this study, nearly all the midwives interviewed have mentioned the cost of hospital delivery as a main factor that hinders women with obstetric complications from using a hospital. The majority of the midwives interviewed explained the issue using the following expression or one similar to it:

"Cost of services in hospitals is a major barrier to seeking care" [Midwives 2].

A TBA addressed the issue this way:

"When I told that she must been taken to a hospital, her husband sat and cried that I do not have money" [TBA 1].

Formerly, Iran had a special health-insurance programme called *"at-bed insurance," *designed to make medical services affordable for all people. When Iranians arrived at a hospital without insurance, they could use this coverage for hospital costs. The patient would pay 10% of the costs. The insurer would pay the rest. However, this programme has been replaced by "*self-paid insurance*", which costs 400,000 IRR (approximately US$ 30) for each family member per year. Additionally, one mother noted the following:

"Further financial aids are available for special cases such as those whose husbands are in prison and their expenses are paid by penitentiaries' administration" [Mother 1].

However, in Zahedan, this programme has a number of problems. First, because Zahedan families often include many children, sometimes they cannot afford the insurance premium. One TBA noted the following:

"When they have 5 or more children, the amount of premium is approximately equal to the cost of hospital delivery and it is unaffordable for them to buy insurance" [TBA 1].

If a family was unable to pay the premium, with the approval of the *Imam Khomeini Relief Foundation (IKRF)*, the family can receive up to a 100 per cent discount. However, especially in the suburban and disadvantaged districts of the city, because many women do not have an Iranian national ID card or passport, they cannot take advantage of this fee-exemption programme. A number of midwives noted this issue in the following way:

"There are two problems with this programme in Zahedan. First, some of Baluch's women and families don't have any information about this insurance; and second, especially in the suburban and disadvantaged districts of the city, many women don't have an ID card, and can't accordingly make use of this opportunity" [Midwife 5, 6, 9].

The interviews and observations cited above provide ample evidence that financial inaccessibility, which stems from interconnected social problems, is one of the main barriers to referring Baluch women with obstetric complications to hospitals.

### The perceived quality of obstetric care

The reluctance to use EmOC can also be ascribed to people's assessment of the quality of such care. A number of midwives noted as follows:

"when we talk about referring to women, the woman's family immediately answers us that it's better she dies here than goes to hospital" [Midwife 4, 9, 11].

In Zahedan, as some midwives commented, "Fear of Caesarean section and some kinds of procedures like curettage is an obstacle for women and families to use the hospital" [Midwife 4, 6].

Mothers had problems understanding the rationale behind Caesarean section and the procedure's wide prevalence in hospitals (see Table 3). Baluch mothers stated that doctors subjected them to Caesarean section without an acceptable reason.

**Table 3:** Caesarean section as a proportion of all births in Zahedan

Number of C/S	Expected number births in Zahedan	Proportion Zahedan/hospitals		Recommended range
2501	15150	0.17	0.21	5-15%

Source: MHO, Zahedan, 2011.

"In hospitals, doctors are ready for Caesarean, if the child birth lasts only for a short longer time; they will soon precede an operation" [Mother 2, 7, 15].

As a result, Baluch women postpone transferring to hospitals and persistently seek to give birth normally at home. Even when told by biomedically educated midwives that transferring to hospital was necessary, the women called a TBA to ensure that normal vaginal delivery is not possible.

At the same time, the midwives believed that a main reason for delay by women and their families is as follows:

"After referring women to hospitals, physicians hurry in decision making; and most of the time they offer women Caesarean sections" [Midwife 4].

In accordance with the above quotation, one midwife interviewed noted the following:

"Personal midwife must accompany women to hospital and stay with her in hospital" [Midwife 2].

Another midwife commented in the following way: "it is necessary to produce a sense of safety for patients, and in this way midwives can convince their patients more easily to come to hospital in complicated cases" [Midwife 3].

In addition, nearly all midwives who live and work in Zahedan stated the following or used a similar expression:

"Absence of the personal midwife in the delivery room, whom the woman trusts, is a barrier to referral" [Midwife 2, 3, 10, 12].

### Socio-cultural deterrents

As Table 4 reveals, 77% of all births in Zahedan occur in hospitals, while the proportion for the entire urban area of Sistan and Baluchestan province is 60.86%. That is, 39.14% of all births in the province occur at home. The evidence also shows that 23% of all deliveries that occur in non-hospital settings and 75.16% of home births in Zahedan had been supervised by TBAs.

**Table 4:** Proportion of births in available comprehensive EmOC public facilities in Zahedan

Facility type	Number of births	Expected number of births	Proportion	Recommended
4 comprehensive EmOC facilities	11.754**	15150*	0.77	>15%

*To calculate this number, we used the total number of annual births in Zahedan according to the Maternal Health Office, Zahedan, 2011.

**This number summarises Caesarean sections and normal vaginal deliveries in three comprehensive EmOC facilities.

Source: MHO, Zahedan, 2011.

The qualitative data of this study indicated that major socio-cultural factors were contributing to the commonness and rationalisation of home childbirth among Baluch families in Zahedan. When we asked participant midwives why Baluch women do not want to go to hospitals even when they have complications, their answers referred primarily to delays in decision making. As some biomedically educated midwives noted: "referral depends on various factors, and delays take place because we need time to convince women and their families to go hospital and use emergency obstetric-care facilities" [Midwife 6].

Understanding the reasons that women and their families have for delaying the decision to go to a hospital is important for grasping the main aspects of the problem. Sometimes, the delay is related to people's beliefs about the causes and remedies of disease. For example, in Sistan and Baluchestan province, convulsions may be associated with supernatural causes that are believed to require exorcism by faith healers rather than medical attention. However, one TBA interviewed stated the following:

"But nowadays these issues hardly occur, and in my opinion, this belief belongs to the past" [TBA 2].

Nevertheless, the notion of disgrace was among the most important factors that can cause a delay of the first type. This idea refers to a mark of infamy or a label that is often difficult to hide in a community [21]. One biomedically educated midwife characterised the significance of this type of disgrace as follows:

"When I arrived there was an untrained TBA. She sat in front of the parturition mother and did not allow me to examine her, and although the relatives knew that something was wrong, no one did anything against TBA" [Midwife 2].

Another mother told us about her sister's delivery. She said,

"When I went in my sister's home she did not feel well at all. She had been suffering the labour pain since the previous night and had not yet given the birth. When I was trying to take her to hospital, my mother asked me to be careful not to say something which might resent the TBA (Mother 3).

When the study's main author asked this woman's mother "why did not you do anything against TBA, while you felt something was wrong, and your daughter's life was in danger?" she instantly replied as follows:

"It was impossible because I was afraid that TBA would go outside the home and shout and bring disgrace on us" [Mother 17].

Finally, to save the mother, a neighbour had begun to threaten the TBA and the advice that the mother transfer to a hospital. However, the mother's family had had to pay the TBA and drive her home before the mother could be taken to a hospital.

A Baluch biomedically educated midwife explained the passivity of the mothers' reactions accordingly:

"Other family members, relatives, and neighbours refer to TBAs for different reasons (delivery, prenatal cares, oiling and massaging the abdomen which they believe will facilitate delivery and occasionally will help the external rotation of the foetus); so the TBAs are likely to start backbiting about the behaviour of mothers who had not respected them and develop gossips about those mothers and their families. So mothers, to avoid such situations, have to take tolerant behaviour with the TBAs. Moreover, in case mothers and their family needed the TBA, she would not help them anymore (Midwife 4).

The following mother's story confirms how Baluch mothers are victimised by this type of disgrace:

"I am afraid of people's words or labels about me or my family. It would be very bad for you. You would easily be disgraced, no one would respect you; you would be then depressed and lonely" (Mother 6)

Consequently, this type of disgrace and similar social phenomena can cause mothers and families in the Baluch community to delay when considering transferring to hospitals.

### Professional relationships between midwives and physicians

Another aspect of the problem relates to professional relationships between the midwives and the hospital obstetricians. Collaboration, mutual recognition and satisfactory professional relationships between the two groups are central to hospital efforts to provide competent maternity and obstetric care. Such competence is hard to achieve [22-24]. A number of midwives reported that professional rivalries between midwives and doctors and misbehaviour by physicians and their colleagues in hospitals sometimes contribute to the problem of delayed referral acceptance.

According to the data, gynaecologists and obstetricians do not trust midwives. This mistrust can inhibit mutually satisfying professional relationships from developing between midwives and physicians. Additionally, midwives lack the assurance that the medical team will support them at critical moments. They noted as follows:

"Hospitals and physicians do not cooperate and they aren't supportive; they even urge the mother's relatives to complain and sue us" [Midwife 5].

Although TBAs supervise 75% of deliveries in Zahedan, they are not legally allowed to supervise deliveries in urban areas. As a result, they can easily be sued. Therefore, the TBAs endeavour to complete the deliveries at home and do not refer parturient mothers to hospitals except in the case of a serious complication. In addition, although delivery at home supervised by a biomedically educated midwife is fully legal, these midwives are also hesitant to refer mothers to hospitals. Some biomedically educated midwives presented their reasons as follows:

"Physicians inquire and blame us in the presence of the patient and her relatives [Midwife 9], and "They don't take into account and respect us" [Midwife 1].

Therefore, physician and hospital staff behaviour can be a threat to the midwife's job and prestige. One midwife stated the following:

"If midwives refer a woman to the hospital, the woman and her family think she is not competent, and she will lose all credibility in the eyes of the patient" [Midwife 4, 8].

## Discussion

The study reveals that several relatively distinct processes are involved in the creation of the circumstances that lead to indecisiveness and delay in referral by the midwives and referral acceptance by the mothers when life-threatening complications arise. These deterring processes or reasons are as follows: socio-cultural barriers and the economic inaccessibility of EmOC; professional relationships between midwives and physicians and perceived quality of care at EmOC facilities.

A close examination of the literature revealed that EmOC use is not merely a question of availability [25] but also of financial accessibility [26]. Income and health insurance make services affordable and, consequently, financially accessible. However, financial affordability does influence the use of comprehensive EmOC [5,7] by Baluch women. Several studies call attention to the important links between service delivery and the macro-dynamics of a health-care system's structure and financing. Such studies remind policy makers and managers that a health-care system is a socio-cultural institution and that a community's health-care problems cannot be addressed without first addressing these relationships, including the socio-cultural structure on which these relationships are frequently based [27].

The present study showed that bureaucratic obstacles among Baluch women such as the lack of a national ID card or passport can cause inequity in access to governmental facilities and strategies for reducing the financial barriers to emergency obstetrical care, e.g., insurance and insurance discounts. Some scholars concluded that a targeted fee-exemption programme is necessary for poor or otherwise disadvantaged mothers (those with no national ID card or those with large families and limited financial resources). However, other studies showed that making services accessible might not be enough [28] because socio-cultural factors can inhibit the use of EmOC facilities despite financial accessibility. Therefore, this study verifies that biomedical models discount the role of socio-cultural factors in determining care-seeking behaviour [29].

Research has shown that the relationship between potential users, the socio-cultural structure, and health beliefs affect the use of health-care services such as comprehensive EmOC [30,31]. Traditionally, risk definitions have focused on the likelihood that an unexpected event will occur [32]. As a result, the traditional approach to risk reduction has been concerned with managing risk through applying medical sciencetific advances in highly specialised areas [33]. Recently, however, social scientists have rejected the notion of real or objective risk. They believe that an individual's own estimate of risk may differ greatly from an objective estimate of that risk. Perceived risk concerns how an individual understands and experiences the phenomenon. Furthermore, risk is a socio-cultural construction [34].

To understand why parturient mothers and their families in Zahedan react passively to hospital referrals by TBAs and why TBAs did not refer mothers to EmOC as soon as the TBAs perceived a serious delivery complication, we must first understand the concept of stigma and the process of stigmatisation (21). The local society teaches the rules that organise interpersonal life by immersing the members of the society in established cultural patterns. When a disparity occurs between an individual's "virtual social identity", that is, how a society characterises an individual, and the individual's "actual social identity", or the attributes the individual actually possesses, the individual's identity may be stained. The individual is reduced from a whole and ordinary person to a tainted and improper one who runs the risk of stigmatisation [35]. Stigmatisation threatens what is most at stake for ordinary people in a local society such as their relationships with others and their life chances [36]. As the mothers in this study showed in their interviews, these individuals face the severe social sanction of isolation or marginalisation from their local world. Sometimes, the individual and the individual's family experience a dread that is felt more strongly than physical fear [37]. Therefore, as in the behaviour of one mother's family cited above, who gave money to a TBA and showed respect to her, community members try to fulfil the obligations of reciprocity and show consideration for the feelings of others to maintain and reinforce their moral reputation in the local society and prevent potential ostracism [37].

The findings of the present study are consistent with Berry (2006), who showed that women and families prioritised social variables over biological ones. This result shows that Zahedan women and families use their own criteria to assess home-birth emergencies and the necessity of EmOC in a manner that sometimes differs from medical guidelines [38]. This behaviour arises from contextual differences in the meaning and use of the concept of risk [39] and the fear of stigmatisation [40]. A model with two dimensions, an objective or scientific risk and a socially experienced or lived risk, has been introduced to explain the differing risk concepts. While epidemiologists use the objective or scientific dimension to interpret risk, laypeople use social or lived experiences [39]. In the general population, risk can be internalised and experienced as an immeasurable insecurity, a system that punishes and rewards individual behaviour in a way that is accepted and desired. However, what is acceptable in one society would be banned in other societies and cultures. Violating social rules can result in exclusion from the community [41]. The present study is consistent with Murphy (2011), who showed that beyond the idea of the loss of individuality, issues of identity might be at stake for ordinary people. The present study also showed that socio-cultural risks, including the threat of social exclusion, are sometimes more important than medical risks, which can delay care-seeking.

The present study accentuates the key role of the midwife in encouraging or discouraging mothers and their families to accept or decline the referral to comprehensive EmOC [42]. Likewise, the present study showed that midwives may fail to refer patients to hospital-based services because the midwives are unable to address the interpersonal aspects of the referral circumstances. On the other hand, when patients were reluctant to transfer, these nurses lacked the knowledge and communication skills to overcome this reluctance [7,43]. Referral decisions are not just matters of technical or organisational considerations. The decisions also involve stress, fear, and anxiety on the part of the mothers and their families and the midwife. The present study showed that poor professional interactions between frontline midwives and hospital staff and physicians can threaten the professional prestige of midwives. The results of the present study and those of another study showed that the failure to adequately refer patients can originate in the negative attitude towards TBAs and biomedically educated midwives of hospital staff and the TBA's and biomedically educated midwive's fear of losing credibility [43]. As Yung (2007) noted, these fears can threaten a midwife's socioeconomic status, income, life chances, good fortune, job, local social network and relationships with colleagues for biomedically educated midwife. Consequently, "midwives prefer to keep their patients 'under observation' at home as long as possible, often beyond what is reasonable. They only considered a referral when surgery becomes inevitable or they can't do anything else" [43]. Therefore, poor cooperation and interpersonal relationships between physicians and midwives can cause under use of these facilities even where the facilities are readily available.

Additionally, as the literature reviews recognise [5,6], the assessment of the quality of obstetric care plays a key role in the decision to seek hospital treatment. This assessment depends largely on an individual's experience with the health-care system and the experiences of acquaintances. The literature showed that the unique understanding that women have of their pregnancies and births leads them to different evaluations from those of men and health-care professionals of what constitutes an obstetric emergency and the best manner to address such an emergency [38,44]. The way women understand pregnancy and childbirth in their everyday lives encourages them and their families to think and behave differently from what health guidelines would dictate. Therefore, policy makers and managers must be aware that acceptance and sustained use of health services depends on women's views on the quality of services. Because childbirth is a culturally and emotionally sensitive area, quality of services should address user and provider satisfaction, social, emotional, medical and financial outcomes as well as aspects of equity and performance according to standards and guidelines [45]. In the present study, some midwives explained that a contract between midwives and hospitals would solve many problems. Furthermore, because midwives are the bridge between hospitals and community members who choose home birth, good communication, trust and satisfying collaboration among midwives, obstetricians, hospitals authorities, families and relatives are necessary for an effective referral system.

The limitations of the current study include the relatively small sample group of midwives, TBAs and mothers who were recruited from Zahedan. The study may reflect a unique set of circumstances within the main city of the province. Therefore, no claims are made for the wider applicability of the findings. Further inquiries on the subject with accounts and narratives from other relevant and professional groups or stakeholders, including family physicians and obstetricians, are required to comprehensively explore the issues raised. Notwithstanding these limitations, the study's findings offer valuable insight into the lived experiences of mothers, biomedically educated midwives and TBAs, the barriers to referring Baluch women with obstetric complications to hospitals, and above all the socio-cultural construction of the hindering factors.

## Conclusion

Consistent with other relevant research, the present study showed the effects of certain socio-cultural, economic, professional and organisational factors on care-seeking behaviour [46]. The evidence confirms that the use of maternity services is not the same in all socio-cultural and political contexts. In accordance with the literature [2,8], this study suggests that significant or influential individuals including mothers-in-law, grandmothers, husbands, other important relatives and community members, and primary-level health-care providers play key roles in making referral decisions about sending parturient women to hospitals during life-threatening complications. This study supports the results of other relevant studies in the literature [38] that find that as long as mothers, their families and midwives use criteria other than health-system guidelines to evaluate when to accept referral to hospitals or, respectively, to make the referral, the referral system is challengeable.

In all, understanding how moral experience and the threat of stigmatisation affect local social life can effectively contribute to increasing the acceptability of referral in cases of life-threatening complications. In addition, hospitals can become more secure, amenable and attractive environments for giving birth by improving their practices and services and taking into consideration the ethical concerns and cultural considerations of Iranian Baluch Therefore, poor cooperation and interpersonal relationships between physicians and midwives can cause under use of these facilities even where the facilities are readily available. The appropriate management of patient charges by hospital authorities and the implementation of a target-fee exemption programme in the case of especially disadvantaged mothers can help mothers and midwives make a rational decision when life-threatening complications arise.

## Abbreviations

EmOC: Emergency Obstetric Care; MM: Mixed Methods; TBA: Traditional Birth Attendants; IMES: Information Management Evaluation System Ministry of Health & Medical Education of Iran; MHO: Maternal Health Office/MHO Zahedan University of Medical Sciences: Zahedan; WHO: World Health Organisation; PMMN: Prevention of Maternal Mortality Network; IKRF: Imam Khomeini Relief Foundation.

## Competing interests

The authors declare that they have no competing interests.

## Authors' contributions

All authors (MGT, ZM, and AAV) participated in planning, conceptualising the research questions, designing the study, managing and interpreting the data and drafting the manuscript. ZM was responsible for organising the field activities and retrieving data in the field. All authors contributed to interpreting the evidence and critically revising the manuscript.
